# Neuroprotective Properties of the Standardized Extract from *Camellia sinensis* (Green Tea) and Its Main Bioactive Components, Epicatechin and Epigallocatechin Gallate, in the 6-OHDA Model of Parkinson's Disease

**DOI:** 10.1155/2015/161092

**Published:** 2015-06-18

**Authors:** Natália Bitu Pinto, Bruno da Silva Alexandre, Kelly Rose Tavares Neves, Aline Holanda Silva, Luzia Kalyne A. M. Leal, Glauce S. B. Viana

**Affiliations:** ^1^Faculty of Medicine of the Federal University of Ceará, Rua Nunes de Melo 1127 (Rodolfo Teófilo), 60430-270 Fortaleza, CE, Brazil; ^2^Faculty of Medicine Estácio of Juazeiro do Norte, Avenida Tenente Raimundo Rocha 515 (Cidade Universitária), 63048-080 Juazeiro do Norte, CE, Brazil

## Abstract

*Camellia sinensis* (green tea) is largely consumed, mainly in Asia. It possesses several biological effects such as antioxidant and anti-inflammatory properties. The objectives were to investigate the neuroprotective actions of the standardized extract (CS), epicatechin (EC) and epigallocatechin gallate (EGCG), on a model of Parkinson's disease. Male Wistar rats were divided into SO (sham-operated controls), untreated 6-OHDA-lesioned and 6-OHDA-lesioned treated for 2 weeks with CS (25, 50, or 100 mg/kg), EC (10 mg/kg), or EGCG (10 mg/kg) groups. One hour after the last administration, animals were submitted to behavioral tests and euthanized and their striata and hippocampi were dissected for neurochemical (DA, DOPAC, and HVA) and antioxidant activity determinations, as well as immunohistochemistry evaluations (TH, COX-2, and iNOS). The results showed that CS and catechins reverted behavioral changes, indicating neuroprotection manifested as decreased rotational behavior, increased locomotor activity, antidepressive effects, and improvement of cognitive dysfunction, as compared to the untreated 6-OHDA-lesioned group. Besides, CS, EP, and EGCG reversed the striatal oxidative stress and immunohistochemistry alterations. These results show that the neuroprotective effects of CS and its catechins are probably and in great part due to its powerful antioxidant and anti-inflammatory properties, pointing out their potential for the prevention and treatment of PD.

## 1. Introduction

Green tea has attracted significant attention worldwide for its benefits for a varied number of disorders, ranging from cancer to weight loss [1]. Although there are several polyphenolic catechins in green tea, epigallocatechin-3-gallate is the most abundant one, accounting for 65% of its total catechin content [2], being probably responsible for most of green tea medicinal properties. Green tea and its bioactive constituents are best known for their antioxidant properties, leading to clinical studies in diseases associated with reactive oxygen species, such as cancer or cardiovascular and neurodegenerative diseases [3–9].

Evidences [10–12], including those from our laboratory [13], have indicated the anti-inflammatory properties of green tea and epigallocatechin gallate. Furthermore, inflammation has been implicated in neurodegenerative pathologies, as Parkinson's disease [14–16]. Parkinson's disease is the second most common neurodegenerative disorder after Alzheimer's disease. It is characterized by a slow and progressive degeneration of dopaminergic neurons in the* substantia nigra pars compacta*. Although the cause of this neuronal degeneration may be poorly understood, it is largely accepted that neuroinflammatory mechanisms are certainly involved [14–22].

In addition, oxidative stress is shown to be associated with neuronal degeneration, as shown in previous [23, 24] and more recent works [25–30]. The oxidative stress contributes to the cascade leading to dopamine cell degeneration in PD and is closely linked to other components of neurodegeneration, as mitochondrial dysfunction, excitotoxicity, increased oxygen free radical production, and inflammation, as well [31]. An important consequence of oxidative stress is an increased lipid peroxidation found in the* substantia nigra* of PD patients [23].

Polyphenols, such as epigallocatechin gallate, due to their anti-inflammatory and antioxidant effects, are known to present neuroprotective properties, what could explain their benefits in neurodegenerative diseases [32–36]. Although there are some data in the literature on the effects of green tea and its polyphenols on Parkinson's disease, the great majority of them deal with* in vitro* models [37–39] or studied the beneficial effects of green tea consumption by Parkinson's disease patients [39].

Thus, the objectives of the present work were to evaluate the neuroprotective properties of the standardized extract of green tea and its catechins on the 6-OHDA model of Parkinson's disease in rats. For that, behavioral evaluations (apomorphine-induced rotation, open field, water maze, rota rod, and forced swimming tests) and neurochemical determinations (striatal DA, DOPAC and HVA measurements, and antioxidant activity) were performed. Besides, immunohistochemistry assays for TH (dopaminergic degeneration marker) and for COX-2 and iNOS (inflammation-related enzyme) were also carried out in 6-OHDA-lesioned animals, without and with drugs treatments.

## 2. Material and Methods

### 2.1. Drugs and Reagents

6-hydroxydopamine, apomorphine, and HPLC standards were from Sigma-Aldrich (St. Louis, MO, USA); ketamine and xylazine were from König (Santana de Parnaíba, São Paulo, Brazil). Antibodies for immunohistochemistry assays were from Santa Cruz Biotechnology (Dallas, TX, USA) or Merck-Millipore (Darmstadt, Germany). All other reagents were of analytical grade.

### 2.2. Animals

Male Wistar rats (200–250 g) from the Animal House of the Faculty of Medicine Estácio of Juazeiro do Norte were maintained at a 24 ± 2°C temperature, in a 12 h dark/12 h light cycle, with standard food and water* ad libitum*. The study was submitted to the Ethical Committee for Animal Experimentation of the Faculty of Medicine of the Federal University of Ceará (Brazil) and was approved under the number 104/2011. All experiments followed the ethical principles established in the Guide for the Care and Use of Laboratory Animals, USA, 1986.

### 2.3. Determination of the Total Phenol Content in the Dried Extract from* C. sinensis*

Samples (10 mg) of the extract were transferred to a 10 mL volumetric flask, containing 0.25 mL 1 N Folin-Ciocalteau reagent and 4 mL Milli-Q water. After alkalinization of the medium (1.25 mL 20% Na_2_CO_3_ solution), the volume was completed to 10 mL with Milli-Q water. After 40 min, at room temperature (25°C) and at dark, the mixture was submitted to spectrophotometric reading at 715 nm [40]. The calibration curve was prepared with standard gallic acid (dissolved in Milli-Q water), at concentrations ranging from 2 to 16 *μ*g/mL. The results showed 32.6% total phenols in the* C. sinensis* extract, determined as gallic acid equivalents.

### 2.4. Experimental Protocol Used for the 6-OHDA Model of PD

The animals were anesthetized with an association of xylazine (10 mg/kg, i.p.) and ketamine (80 mg/kg, i.p.), submitted to trichotomy of the head superior region, and fixed to the stereotaxic frame by their ear canals. Then, a longitudinal midline incision was done and tissues were separated for bregma visualization. The following coordinates (at two different points from bregma) were used: 1st point (AP, +0.5; ML, −2.5; and DV, −5.0) and 2nd point (AP, −0.9; ML, −3.7; and DV, +6.5) according to Paxinos and Watson, 1997 [41]. Then, a thin hole was performed in the skull, over the target area, and a 1 *μ*L solution containing 6 *μ*g 6-OHDA was injected into each point. The syringe stayed in place for 5 min to assure the solution diffusion, and then the incision was sutured. The sham-operated (SO) animals were subjected to all procedures, except that saline was injected into the two points. Afterwards, the animals returned to their cages for recovering. They were divided into the following groups: SO (treated by gavage with distilled water); 6-OHDA-lesioned (also treated by gavage with distilled water); 6-OHDA-lesioned and treated with 25 or 50 mg/kg of the standardized extract of* C. sinensis* (CS25 and CS50); epicatechin, 10 mg/kg (EC10); or epigallocatechin gallate, 10 mg/kg (EGCG10). All treatments started 1 h before the stereotaxic surgery and continued daily for 15 days, with drug volumes of 0.2 mL/100 g body weight. Then, after treatments and 1 h after the last drug administration, the animals were submitted to behavioral tests. At the next day, they were euthanized (decapitation) and brain tissues were removed for neurochemical, histological, and immunohistochemical studies.

### 2.5. Behavioral Testing

#### 2.5.1. Apomorphine-Induced Rotations

Contralateral rotations (opposite to the lesioned right-side) induced by apomorphine (1 mg/kg, i.p.) were monitored for 1 h. The cause of this apomorphine-induced rotational behavior is related to the imbalance, in the nigrostriatal dopaminergic pathways, between the right (lesioned) and left (unlesioned) brain hemispheres. All groups were treated for two weeks. The SO and untreated-6-OHDA groups were administered with distilled water, for the same period of time.

#### 2.5.2. Open Field Test

This test evaluates a stimulant or depressant drug activity and may also indicate an anxiolytic action. The arena was made of wood, whose dimensions were 50 cm × 50 cm × 30 cm (length, width, and height). The floor was divided into 4 quadrants of equal size. At the time of the experiment, the apparatus was illuminated by a red light. The number of crossings with the four paws from one quadrant to another (what measures the locomotor spontaneous activity) was determined.

#### 2.5.3. Forced Swimming Test

The test is based on the observation that when the animals are subjected to a stressful situation with no possibility for escaping, they adopt a posture of immobility after an initial period of agitation. The reduction of this immobility time is suggestive of an antidepressant action. The animals were placed individually in a cylinder (40 cm height and 23 cm diameter), containing water up to 25 cm below the top. The immobility time was monitored for 5 min.

#### 2.5.4. Water Maze Test

The water maze consisted of a black circular pool (1.7 m diameter and 1 m height) with water (0.59 m deep) at 22°C, and visual cues on the walls at one of four equally spaced locations: north (N), east (E), south (S), and west (W). The pool was divided into 4 quadrants: NW, NE, SE, and SW. After the treatment, each animal was submitted to two trials in the pool, for the next three days. In these trials, the animals were placed into the pool, facing the wall, and had a maximum time of 54 s to find a submersed platform. The animal stayed on the platform for 10 s. Animals that did not find the platform at the end of 54 s were placed on it for 10 s. After this time, the animal was removed from the pool for 30 s, and the procedure was repeated (6 times for each animal). The procedure was repeated again 24 h later (pretest). After 48 h of the pretest, the procedure was repeated once more for evaluating the time needed by the animal to find the platform; this correlates with the animal's spatial memory learning ability.

### 2.6. Oxidative Stress Evaluation

#### 2.6.1. MDA Determination

The degree of lipoperoxidation was measured by means of the thiobarbituric acid reactive substances (TBARS) method [42]. For that, 10% striatal homogenates from all groups studied were prepared in 1.15% KCl. Then, 0.25 mL of each homogenate was added to 1 mL 10% trichloroacetic acid and 1 mL 0.6% thiobarbituric acid. After agitation, the mixture was maintained in water bath (95–100°C) for 15 min, followed by the addition of* n*-butanol (2 : 1, v/v). The mixture was cooled in an ice bath and centrifuged (800 ×g, 5 min). The TBARS content in the supernatants was spectrophotometrically determined at 535 nm. The results are expressed in *μ*mol malondialdehyde (MDA) per g tissue.

#### 2.6.2. Nitrite Determination

For nitrite determination, 10% striatal homogenates from all groups studied were prepared in 1.15% KCl. After centrifugation (800 ×g, 10 min), 100 *μ*L of supernatant samples was incubated with 100 *μ*L Griess reagent (1% sulfanilamide in 1% H_3_PO_4_, 0.1% N-naphthylethylenediamide dihydrochloride in 1% H_3_PO_4_) diluted in distilled water (1 : 1 : 1 : 1), at room temperature for 10 min. The absorbance was measured at 550 nm and nitrite concentrations were determined with a standard curve of NaNO_2_ and the results are expressed as *μ*mol nitrite per g tissue [43].

#### 2.6.3. Antioxidant Potential Evaluation by Chemiluminescence in Human Neutrophils

This method is commonly employed for a direct measurement of reactive oxygen species (ROS) generation. It is capable of quantifying both intracellular and extracellular ROS. Briefly, human neutrophils (5 × 10^6^ cells/mL) were incubated, for 20 min at 37°C, with the standardized extract of* C. sinensis* (0.1 to 100 *μ*g/mL) and the luminol chemiluminescent probe (280 *μ*M). After that, the tubes were transferred to the luminometer, and phorbol myristate acetate (PMA, 10^7^ M), as the stimulant reagent, was added. Then, the production of chemiluminescence (CL), in cpm (photons/min), was followed for 20 min at 37°C. The spontaneous production of CL by neutrophils was also performed in the absence of the stimulus, and quercetin (50 *μ*g/mL) was used as standard [44].

### 2.7. Neurochemical Determinations of DA and DOPAC by HPLC

The striatal contents of DA and DOPAC were determined by HPLC. Homogenates were prepared in 10% HClO_4_ and centrifuged at 4°C (15,000 rpm, 15 min). The supernatants were filtered and 20 *μ*L injected into the HPLC column. For that, an electrochemical detector (model L-ECD-6A from Shimadzu, Japan) coupled to a column (Shim-Pak CLC-ODS, 25 cm) with a flux of 0.6 mL/min was employed. A mobile phase was prepared with monohydrated citric acid (150 mM), sodium octyl sulfate (67 mM), 2% tetrahydrofuran, and 4% acetonitrile in deionized water. The mobile phase pH was adjusted to 3.0 with NAOH (10 mM). Monoamines were quantified by comparison with standards, processing the same manner as the samples. The results are expressed as ng/g tissue.

### 2.8. Immunohistochemical Analyses in Rat Brain Areas

#### 2.8.1. Immunohistochemistry Assays for TH, iNOS, and COX-2

Brain striatal or hippocampal sections were fixed in 10% buffered formol, for 24 h, followed by a 70% alcohol solution. The sections were embedded into paraffin wax for slices processing on appropriate glass slides. These were placed in the oven at 58°C, for 10 min, followed by deparaffinization in xylol, rehydration at decreasing alcohol concentrations, and washing in distilled water and PBS (0.1 M sodium phosphate buffer, pH 7.2), for 10 min. The endogenous peroxidase was blocked with a 3% hydrogen peroxide solution, followed by incubation with the appropriate primary anti-antibody for TH, iNOS, and COX-2, and diluted according to the manufacturers' instructions (Santa Cruz or Millipore, USA), for 2 h, at room temperature in a moist chamber. The glass slides were then washed with PBS (3 times, 5 min each) and incubated with the biotinylated secondary antibody, for 1 h, at room temperature in a moist chamber. Then, they were washed again in PBS and incubated with streptavidin-peroxidase, for 30 min, at room temperature in a moist chamber. After another wash in PBS, they were incubated in 0.1% DAB solution (in 3% hydrogen peroxide). Finally, the glass slides were washed in distilled water and counterstained with Mayer's hematoxylin, washed in tap water, dehydrated in alcohol (at increasing concentrations), diaphonized in xylol, and mounted on Entellan for optic microscopy examination. After photomicrographs captures, all data were quantified by the Image J software (National Institute of Health, USA).

### 2.9. Statistical Analyses

For statistical analyses, one-way ANOVA, followed by the Newman-Keuls as the* post hoc* test, was used for multiple comparisons. Whenever needed, the paired or unpaired Student's *t*-test was used, for comparisons between two means. Differences were considered significant at *p* < 0.05.

## 3. Results

### 3.1. Behavioral Evaluation

#### 3.1.1. Apomorphine-Induced Rotations

**Figure 1:**
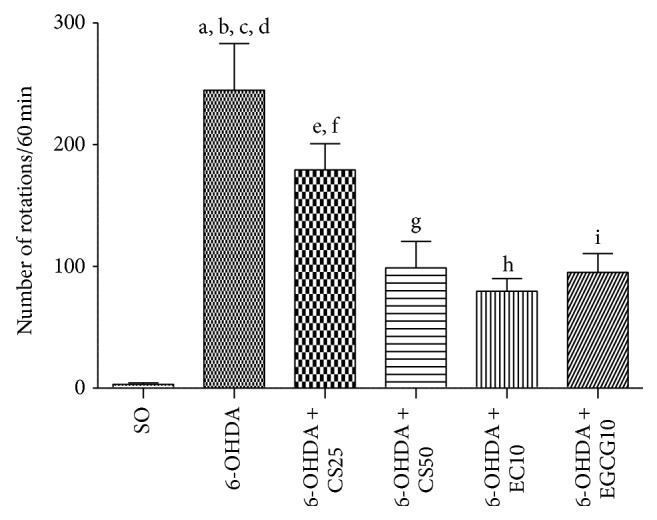
Effects presented by the 6-OHDA-lesioned animals, before and after treatments with CS25, CS50, EC25, or EGCG10, on the apomorphine-induced rotational behavior. a: versus SO, *q* = 11.44; b: versus 6-OHDA + CS25, *q* = 3.19; c: versus 6-OHDA + CS50, *q* = 6.65; d: versus 6-OHDA + EC10, *q* = 7.53; e: versus 6-OHDA + EGCG10, *q* = 6.82; f: versus SO, *q* = 8.97; g: versus SO, *q* = 4.54; h: versus SO, *q* = 3.63; i: versus SO, *q* = 4.36 (one-way ANOVA and Newman-Keuls as the* post hoc* test).

Two weeks after the intrastriatal 6-OHDA lesion, all animals were administered with apomorphine (1 mg/kg, i.p.) and assessed for rotational behavior, for 1 h. A significant increase in the number of contralateral rotations/h was observed in the untreated 6-OHDA group (248.8 ± 38.40), as related to the SO group (3.0 ± 1.43). A partial recovery was exhibited in the 6-OHDA groups after treatments with CS25 (179.4 ± 21.62), CS50 (98.8 ± 21.8), EC10 (79.7 ± 10.40), and EGCG10 (95.2 ± 15.52) (Figure 1).

#### 3.1.2. Open Field Test

**Figure 2:**
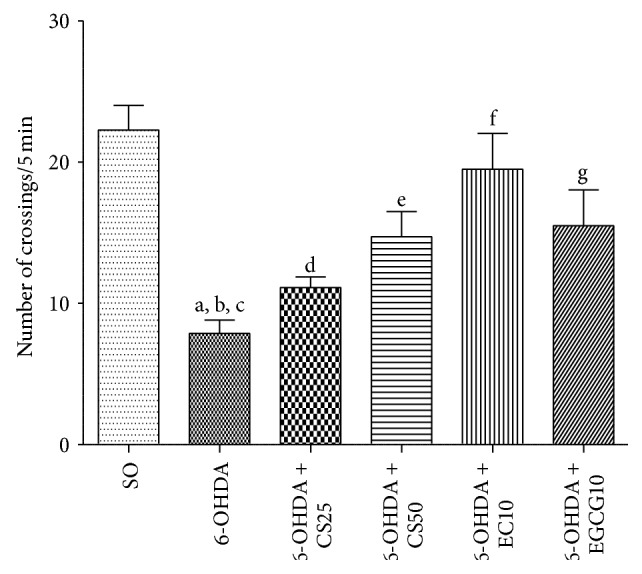
Effects presented by the 6-OHDA-lesioned animals, before and after treatments with CS25, CS50, EC10, or EGCG10, on the locomotor activity as assessed by the open field test. a: versus SO, *q* = 8.75; b: versus 6-OHDA + CS50, *q* = 4.15; c: versus 6-OHDA + EC10, *q* = 6.76; d: versus EGCG10, *q* = 4.44; e: versus SO, *q* = 6.77; f: versus SO, *q* = 4.45; g: versus SO, 3.83 (one-way ANOVA and Newman-Keuls as the* post hoc* test).

The results showed a significant decrease in locomotor activity (65%) in the untreated 6-OHDA group, as related to the SO group. This effect was smaller in the 6-OHDA groups after treatments with the standardized* C. sinensis* extract (CS) that exhibited decreases of 50 and 34%, with the doses of 25 and 50 mg/kg, respectively. Lower decreases were observed in the 6-OHDA group after treatments with EC10 (13%) and EGCG10 (30%) (Figure 2).

#### 3.1.3. Forced Swimming Test

**Figure 3:**
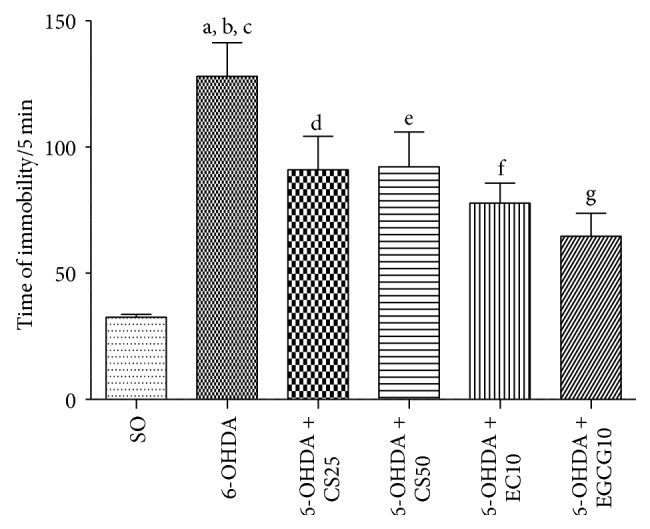
Effects presented by the 6-OHDA-lesioned animals, before and after treatments with CS25, CS50, EC10, or EGCG10, on the immobility time as assessed by the forced swimming test. a: versus SO, *q* = 8.86; b: versus 6-OHDA + EC10, *q* = 4.66; c: versus 6-OHDA + EGCG10, *q* = 5.89; d: versus SO, *q* = 5.07; e: versus SO, *q* = 5.17; f: versus SO, *q* = 3.93 (one-way ANOVA and Newman-Keuls as the* post hoc* test).

The untreated 6-OHDA animals presented a 3.9-fold increase in the immobility time, as related to the SO group, indicating a depressant activity. This alteration was partly reversed after treatments with CS25 and CS50 (around 2.8-fold increase). Similarly, only 2.4- and 2.0-fold increases in the immobility time were observed after treatments of the 6-OHDA lesioned animals with EC10 and EGCG10, respectively (Figure 3).

#### 3.1.4. Water Maze Test

**Figure 4:**
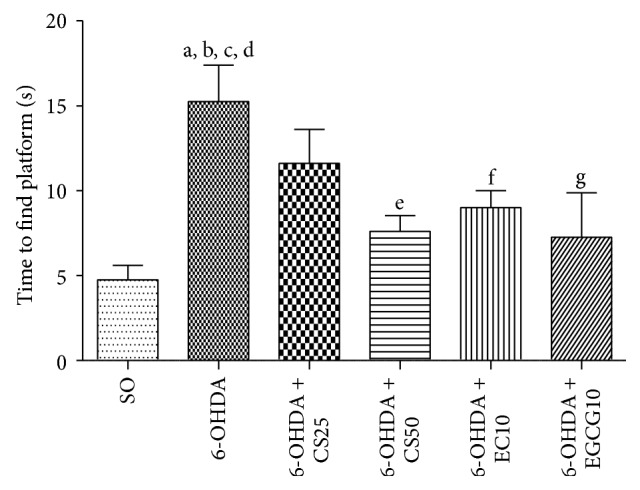
Effects presented by the 6-OHDA-lesioned animals, before and after treatments with CS25, CS50, EC10, or EGCG10, on the time (s) to find the platform as assessed by the water maze test. a: versus SO, *q* = 5.92; b: versus 6-OHDA + CS50, *q* = 4.54; c: versus 6-OHDA + EC10, *q* = 3.71 (one-way ANOVA and Newman-Keuls as the* post hoc* test).

We demonstrated (Figure 4) a 3.2-fold increase in the time to find the platform by the untreated 6-OHDA group, as related to the SO, indicating an alteration of the spacial memory and hippocampal dysfunction. The increase was lower in the 6-OHDA groups after treatments with CS25 and CS50 (2.4- and 1.6-fold, resp.). In the 6-OHDA groups treated with EC10 and EGCG10 the values were close to those of the SO group.

### 3.2. Evaluation of the Oxidative Stress

#### 3.2.1. TBARS Determination

**Figure 5:**
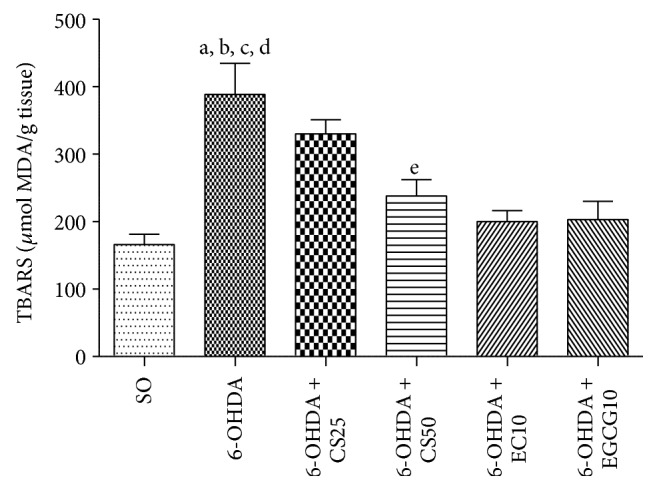
Effects presented by striatal tissues from 6-OHDA-lesioned animals, before and after treatments with CS25, CS50, EC10, or EGCG10, on the lipid peroxidation as determined by the TBARS assay. a: versus SO, *q* = 7.75; b: versus 6-OHDA + CS50, *q* = 5.24; c: versus 6-OHDA + EC10, *q* = 6.89; d: versus 6-OHDA + EGCG10, *q* = 6.77 (one-way ANOVA and Newman-Keuls as the* post hoc* test).

The untreated 6-OHDA group presented a 2.3-fold increase in lipid peroxidation, as related to the SO group. Lower changes (2.0- and 1.4-fold increases) were observed in the 6-OHDA groups after treatments with CS25 and CS50, respectively. Similarly, decreases around 1.2-fold were seen after treatments with EC10 or EGCG10 (Figure 5).

#### 3.2.2. Nitrite Determination

**Figure 6:**
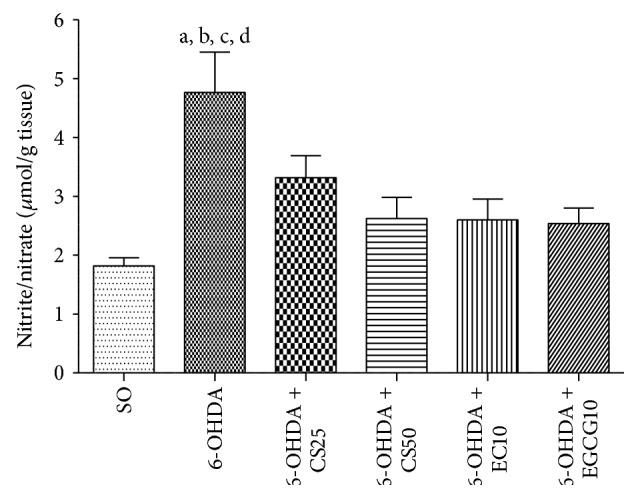
Effects shown by striatal tissues from 6-OHDA-lesioned animals, before and after treatments with CS25, CS50, EC10, or EGCG10, on the nitrite/nitrate contents as determined by the Griess assay. a: versus SO, *q* = 7.09; b: versus 6-OHDA + CS25, *q* = 3.48; c: versus 6-OHDA + CS50, *q* = 4.83; d: versus 6-OHDA + EC10, *q* = 4.89; e: versus 6-OHDA + EGCG10, *q* = 5.36 (one-way ANOVA and Newman-Keuls as the* post hoc* test).

An increase of 2.6-fold in nitrite contents was observed in the untreated 6-OHDA group, as related to the SO group. This increase was of 1.8- and 1.4-fold in the 6-OHDA group, after treatments with CS25 and CS50, and around 1.4-fold after treatments with EC10 or EGCG10 (Figure 6).

#### 3.2.3. Antioxidant Capacity in Human Neutrophils by Chemiluminescence

**Figure 7:**
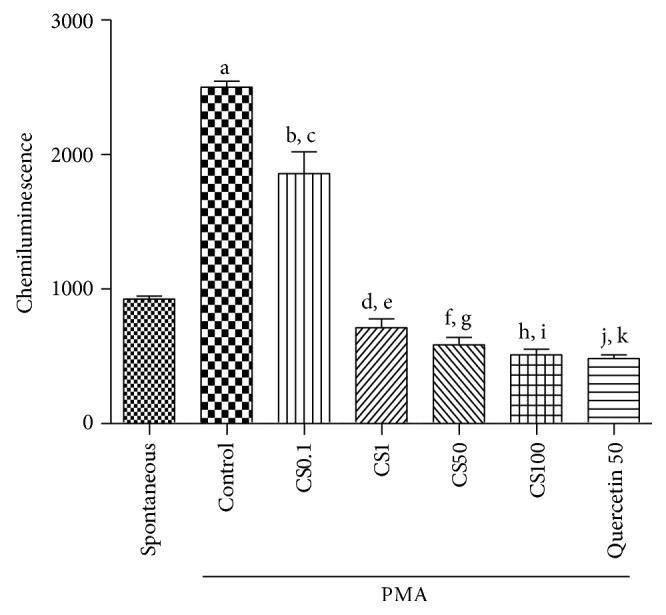
The standardized extract from* Camellia sinensis* (CS), at concentrations ranging from 0.1 to 100 *μ*g/mL, significantly decreased chemiluminescence in human neutrophils, as related to the PMA-stimulated cells, indicative of an antioxidant potential. a: versus spontaneous (spont.), *q* = 25.67; b: versus spont., *q* = 14.09; c: versus spont., *q* = 3.24; d: versus spont., *q* = 5.16; e: versus spont., *q* = 7.25; f: versus control (cont.), *q* = 9,68; g: versus cont., *q* = 27.01; h: versus cont., *q* = 28.93; i: versus cont., *q* = 30.05; j: versus cont., *q* = 32.93 (one-way ANOVA and Newman-Keuls as the* post hoc* test).

An increase of 2.7-fold in oxidative capacity was observed in the controls, after PMA addition, as related to the spontaneous oxidation capacity (Figure 7). Although an increase was also detected with the lower concentration of CS (0.1 *μ*g/mL), it was smaller than that in the presence of PMA. However, the oxidation capacity in the presence of CS decreased by 23, 37, and 45%, as related to controls (spontaneous oxidation), at concentrations of 1, 50, and 100 *μ*g/mL, respectively. This last value was similar to that observed in the presence of quercetin, used as standard (a 48% decrease).

### 3.3. Neurochemical Evaluation

#### 3.3.1. DA, DOPAC, and HVA Determinations in the Rat Striata

**Figure 8:**
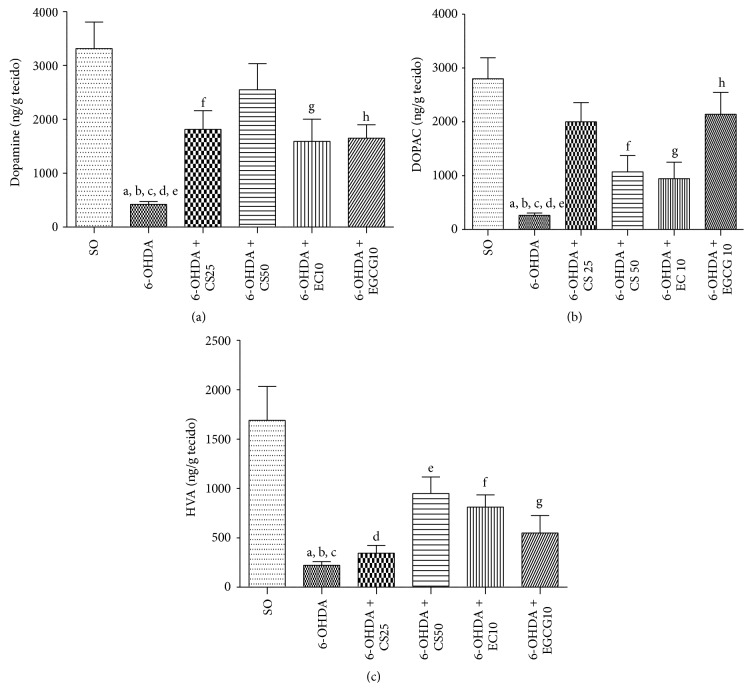
The decreases in striatal DA contents (a), DOPAC (b), and HVA (c) were significantly reversed in the 6-OHDA-lesioned group, after treatments with CS (25 or 50 mg/kg), EC (10 mg/kg), or EGCG (10 mg/kg). (a) a: versus SO, *q* = 8.72; b: versus 6-OHDA + CS25, *q* = 4.99; c: versus 6-OHDA + CS50, *q* = 6.91; d: versus 6-OHDA + EC10, *q* = 3.52; e: versus SO, *q* = 4.38; f: versus SO, *q* = 4.46; g: versus SO, *q* = 4.78. (b) a: versus SO, *q* = 8.69; b: versus 6-OHDA + CS25, *q* = 6.26; c: versus SO, *q* = 5.26; d: versus SO, *q* = 5.27. (c) a: versus SO, *q* = 9.57; b: versus 6-OHDA + CS50, *q* = 5.06; c: versus SO, *q* = 7.71; d: versus SO, *q* = 4.24; e: versus SO, *q* = 4.82; f: versus SO, *q* = 7.10 (one-way ANOVA and Newman-Keuls as the* post hoc* test).

DA striatal depletion in untreated 6-OHDA animals resulted in a decrease of 87%, as related to the SO group. Similar results were observed in DOPAC and HVA contents (91 and 87% decreases, resp.). However, a significant reversion of these effects was demonstrated in the 6-OHDA groups, after treatments with CS25 and CS50, where dose-dependent decreases (45 and 23%) were observed in DA contents. Furthermore, smaller decreases were also seen in DA levels of the 6-OHDA + EC10 (52%) and 6-OHDA + EGCG10 (50%) groups. As far as DOPAC and HVA contents are concerned, significant decreases (62 and 44%) were demonstrated only in the 6-OHDA group, after treatment with the higher CS dose (6-OHDA + CS50), as related to the untreated 6-OHDA group. Similar results were observed with the 6-OHDA + EC10 and 6-OHDA + EGCG10 that presented decreases of 66 and 24% for DOPAC and 52 and 87% decreases for HVA, respectively, as related to the untreated 6-OHDA group (Figures 8(a), 8(b), and 8(c)).

### 3.4. Immunohistochemistry Assays

#### 3.4.1. Effects of Green Tea and Its Catechins on TH, COX-2, and iNOS Immunostainings

**Figure 9:**
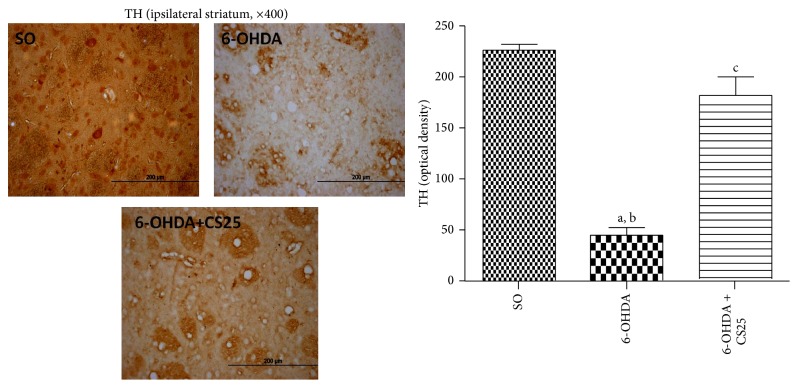
The treatment with the standardized extract of* C. sinensis* (CS) reverses almost completely the depletion of the striatal tyrosine hydroxylase (TH) activity in 6-OHDA-lesioned animals, as evaluated by immunohistochemistry assays. The data were quantified by the Image J software. a: versus SO, *q* = 17.38; b: versus 6-OHDA + CS25, *q* = 12.24; c: versus SO, *q* = 3.85 (one-way ANOVA and Newman-Keuls as the* post hoc* test).

**Figure 10:**
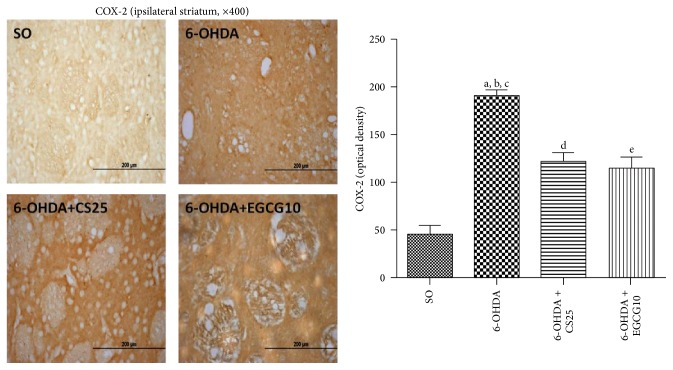
Treatments with CS25 or EGCG10 of 6-OHDA-lesioned animals partly reversed the drastic increase in immunostaining for COX-2 in the striata. The data were quantified by the Image J software. a: versus SO, *q* = 12.27; b: versus 6-OHDA + CS25, *q* = 6.07; c: versus 6-OHDA + EGCG10, *q* = 6.68; d: versus SO, *q* = 7.12; e: versus SO, *q* = 6.47 (one-way ANOVA and Newman-Keuls as the* post hoc* test).

**Figure 11:**
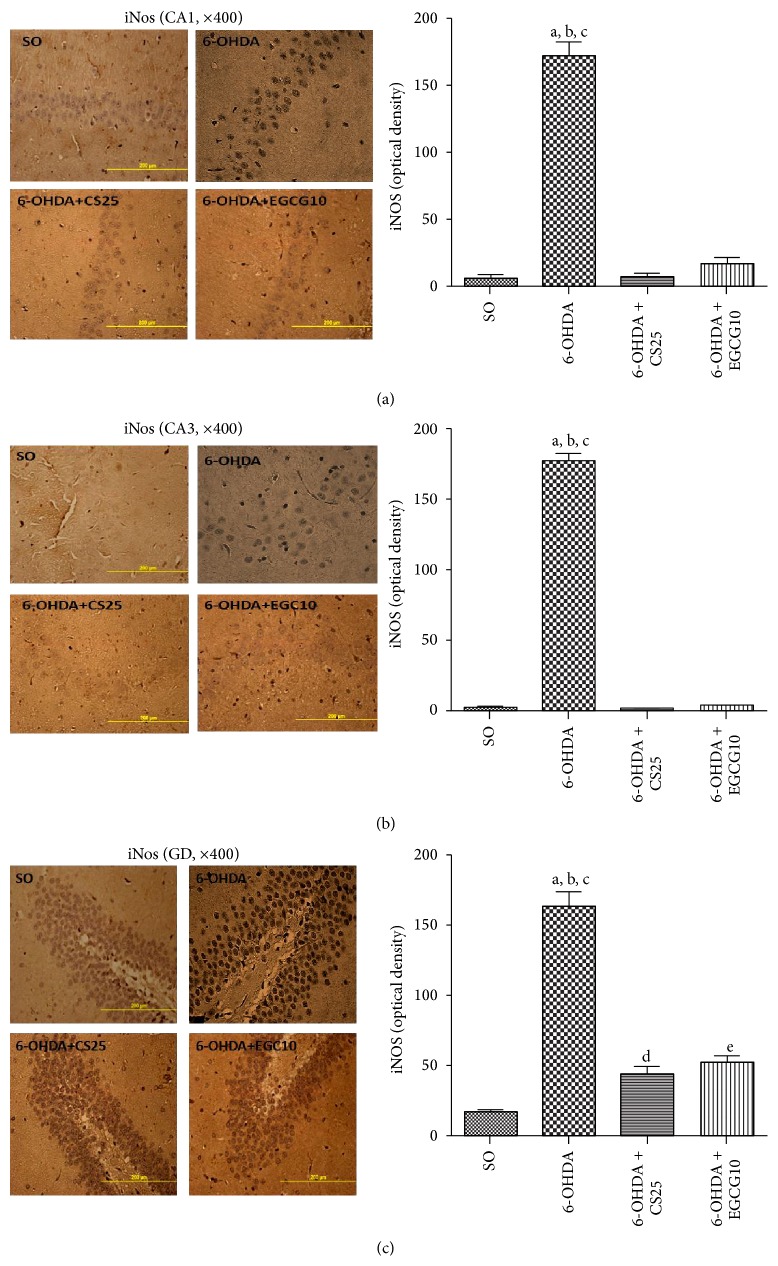
Treatments with CS25 or EGCG10 of 6-OHDA-lesioned animals reversed at least partially the increased immunostainings for iNOS, mainly in CA1, CA3 areas, but also in DG hippocampal subfields. The data were quantified by the Image J software. CA1: a: versus SO, *q* = 23.22; b: versus 6-OHDA + CS25, *q* = 23.26; c: versus 6-OHDA + EGCG10, *q* = 21.82. CA3: a: versus SO, *q* = 74.33; b: versus 6-OHDA + CS25, *q* = 70.31; c: versus 6-OHDA + EGCG10, *q* = 69.67. DG: a: versus SO, *q* = 21.98; b: versus 6-OHDA + CS25, *q* = 17.75; c: versus 6-OHDA + EGCG10, *q* = 15.68; d: versus SO, *q* = 4.01; e: versus SO, *q* = 5.02 (one-way ANOVA and Newman-Keuls as the* post hoc* test).

The untreated 6-OHDA group showed around 80% decreases in striatal immunostaining for TH (Figure 9), as related to the SO group. However, the results observed in the 6-OHDA group after treatment with CS (6-OHDA + CS25) presented immunostaining similar to that observed in the SO group. A 4-fold increase in COX-2 immunostaining (Figure 10) was observed in striatal tissue from untreated 6-OHDA animals, as related to the SO group. In addition, increases of only 2.7- and 2.5-fold were observed in the 6-OHDA groups after treatments with CS25 or EGCG10. Immunohistochemistry assays were also performed for hippocampal tissues in these same groups. The results showed around 24-, 129-, and 10-fold increases in immunostainings for iNOS, in CA1, CA3, and DG subfields in the untreated 6-OHDA animals, as related to the SO group. The values decreased towards those of the SO group in the 6-OHDA group after treatments with CS25 and EGCG10, mainly at CA1 and CA3 areas (Figure 11).

## 4. Discussion

Parkinson's disease (PD) is a chronic, neurodegenerative disorder, involving the degeneration of dopaminergic neurons in the* substantia nigra pars compacta* and leading to the loss of dopaminergic terminals in the striatum. Gait disturbances are among the most important motor problems associated with PD; they occur in all stages of the disorder and are one of the hallmarks for PD progression [45].

*C. sinensis* and its polyphenolic catechins have attracted significant scientific attention for their health benefits in a variety of disorders, including neurodegenerative ones, such as PD [1]. In the present study, we showed that the* C. sinensis* standardized extract and its catechins, epicatechin and epigallocatechin gallate, present a neuroprotective effect on the striatal 6-OHDA model of PD, in rats.

We showed that these drugs reversed, at least partly, the behavioral changes observed in the untreated 6-OHDA-lesioned animals, at very low doses. Thus, in the apomorphine-induced rotational test, significant decreases occurred in the 6-OHDA group after treatments with CS50, as well as with the two catechins. The drugs also reversed the decreased locomotor activity observed in the untreated lesioned group; and similar results were seen with the forced swimming test and water maze test, indicative of antidepressant and spatial learning effects, respectively. Previously [13], we showed that catechin, at small doses (10 and 20 mg/kg), attenuated the increased rotational behavior induced by apomorphine and also the decrease in locomotor activity and working memory deficits, what support our present findings.

Evidences demonstrated that green tea polyphenols possess an antidepressant activity in mice [46], while others [47] showed that several green tea catechins, including EC and EGCG, after a long-term oral administration to rats, improved reference and working memory related to learning ability. All these data agree with ours and point out that these green tea effects could be of benefit in neurodegenerative disorders, as Parkinson's disease.

CS50, EC10, and EGCG10 significantly reversed the decreases in striatal DA, DOPAC, and HVA contents, observed in the untreated lesioned animals. Interestingly, similar effects were seen with all three drugs. Although there are several studies on the neuroprotective effects of green tea and its catechins, most of them concentrate their objective on cellular or* in vitro* models of PD [37, 38, 48–56]. Thus, there are relatively few works dealing with* in vivo* animal models [57–61]. Among these last studies, only that by Levites et al., 2001 [57], investigated the effects of epigallocatechin-3-gallate on the MPTP-induced dopaminergic neuron loss in the* substantia nigra* and striatal dopamine depletion, showing that the pretreatment of mice with the green tea extract or EGCG prevented these effects.

However, in our study, we evaluated not only DA but also DOPAC and HVA contents in the 6-OHDA-lesioned group, after treatments with green tea and its catechins, epicatechin and epigallocatechin gallate. Growing evidences have indicated that oxidative stress and inflammation play a key role in neurodegenerative disorders, including PD, and probably the antioxidant effects of green tea are associated with its neuroprotective action [33]. Supporting this, we demonstrated that green tea and its catechins presented antioxidant effects, as evaluated by the decrease of lipid peroxidation and nitrite contents, observed in striatal tissue from 6-OHDA lesioned animals, after treatments with those drugs. Besides, they present an antioxidant potential in human neutrophils* in vitro*. Others [59] also observed that green tea polyphenols protected dopaminergic neurons, by preventing 6-OHDA-induced increase in ROS and NO levels, lipid peroxidation, and nitrite content, among other effects.

Also, growing evidences from previous and more recent works [11, 50, 62–66] have indicated the antioxidant properties of green tea and its major catechins. Furthermore, it is largely accepted that, in PD, oxidative stress is a common underlying mechanism that leads to cellular dysfunction and demise [24, 30, 31]. ROS are subproducts of mitochondrial respiration and other cellular processes, and some, as superoxide anion, nitric oxide and hydrogen peroxide are essential for redox signaling and cellular function [26].

In the present work, green tea and catechins treatments of 6-OHDA-lesioned animals partly reversed the striatal tyrosine hydroxylase decrease observed in untreated rats, as evaluated by immunohistochemistry assays. Tyrosine hydroxylase is the rate limiting enzyme for DA synthesis in the brain. The mechanisms that control TH gene transcription and TH mRNA translation are also related to the regulation of TH activity. PD is characterized by a severe loss of dopaminergic neurons and depletion of dopamine in the* substantia nigra pars compacta*. Furthermore, reduction of TH expression results in diminished DA synthesis, making this enzyme essential in the pathogenesis of PD, which is considered as a TH-deficiency syndrome of the striatum [67–69].

In addition, we demonstrated that CS and its catechins decrease immunostaining for COX-2, in striatal tissues from 6-OHDA lesioned animals. Unlike COX-1, COX-2 expression is usually minimally expressed in normal tissue, but when activated COX-2 regulates prostaglandin production, primarily within inflammatory cells. This inflammatory response is an important part in healing and repairing. Although some studies also showed that green tea and its catechins decrease COX-2 expression, most of them were performed in human cancer cells [70–73]. Evidences link COX-2 to the progression of PD. Thus, it was demonstrated that the ablation of COX-2 markedly reduced the deleterious effects of MPTP on the nigrostriatal pathway [74]. Besides, COX-2 inhibition prevented the formation of the oxidant species dopamine-quinone, implicated in the pathogenesis of PD [74–76].

Inflammation and oxidative stress are involved in the onset and progression of PD. Thus, evidences have confirmed elevated oxidative stress and inflammatory response occurring early in the disease, contributing to and/or exacerbating the nigrostriatal degeneration [77]. We showed that treatments of 6-OHDA animals with CS25 or EGCG significantly decreased immunostainings for iNOS in rats' hippocampi. Besides, epigallocatechin and epigallocatechin gallate were shown to greatly reduce NO production from the three NOS isoforms, including iNOS [78]. Furthermore, green tea anti-inflammatory and antioxidant properties are certainly justified by its inhibition of proinflammatory enzymes as COX-2 and iNOS.

It is important to notice that in our study we used very low doses of the standardized green tea extract and of its catechins, epicatechin and epicatechin-3-gallate. Furthermore, considering the high rate of green tea consumption worldwide and its potential benefits in several health problems, including neurodegenerative diseases as PD, translational studies should be stimulated, in order to better understand the possible mechanism for the drug neuroprotective action. In this sense, we believe that the drug anti-inflammatory and antioxidant effects are certainly pivotal for its inclusion as a putative candidate in clinics.
